# Glucose Oxidase Loading in Ordered Porous Aluminosilicates: Exploring the Potential of Surface Modification for Electrochemical Glucose Sensing

**DOI:** 10.1021/acs.chemmater.3c01202

**Published:** 2023-09-11

**Authors:** Maximiliano Jesus Jara Fornerod, Alberto Alvarez-Fernandez, Martyna Michalska, Ioannis Papakonstantinou, Stefan Guldin

**Affiliations:** † Department of Chemical Engineering, University College London, Torrington Place, London WC1E 7JE, U.K.; ‡ Department of Electronic & Electrical Engineering, University College London, Torrington Place, London WC1E 7JE, U.K.

## Abstract

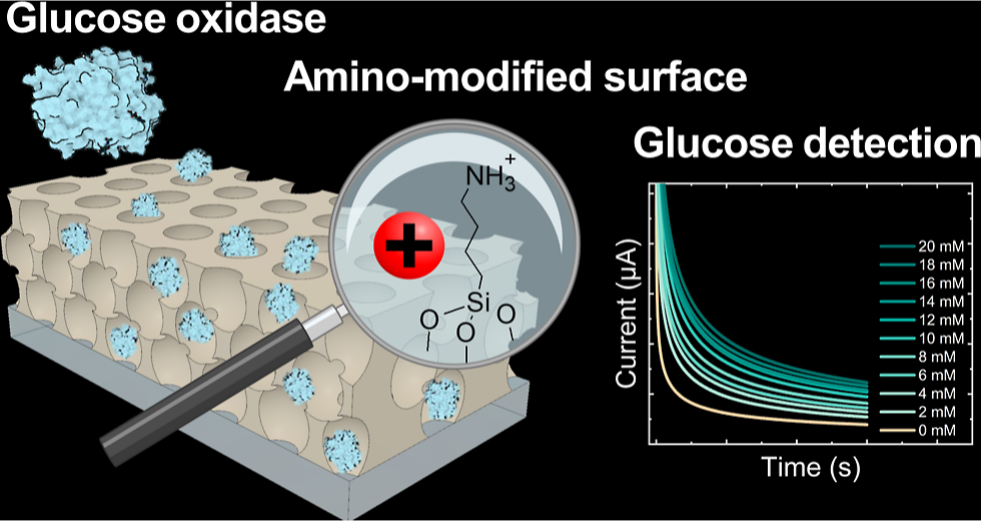

Enzymatic electrochemical sensors have become the leading glucose detection technology due to their rapid response, affordability, portability, selectivity, and sensitivity. However, the performance of these sensors is highly dependent on the surface properties of the electrode material used to store glucose oxidase and its ability to retain enzymatic activity under variable environmental conditions. Mesoporous thin films have recently attracted considerable attention as promising candidates for enzyme storage and activity preservation due to their well-defined nanoarchitecture and tunable surface properties. Herein, we systematically compare pathways for the immobilization of glucose oxidase (GOx) and their effectiveness in electrochemical glucose sensing, following modification protocols that lead to the electrostatic attraction (amino functionalization), covalent bonding (aldehyde functionalization), and electrostatic repulsion (oxygen plasma treatment) of the ordered porous aluminosilicate-coated electrodes. By direct comparison using a quartz crystal microbalance, we demonstrate that glucose oxidase can be loaded in a nanoarchitecture with a pore size of ∼50 nm and pore interconnections of ∼35 nm using the native aluminosilicate surface, as well as after amino or aldehyde surface modification, while oxygen plasma exposure of the native surface inhibits glucose oxidase loading. Despite a variety of routes for enzyme loading, quantitative electrochemical glucose sensing between 0 and 20 mM was only possible when the porous surface was functionalized with amino groups, which we relate to the role of surface chemistry in accessing the underlying substrate. Our results highlight the impact of rational surface modification on electrochemical biosensing performance and demonstrate the potential of tailoring porous nanoarchitecture surfaces for biosensing applications.

## Introduction

Glucose detection and monitoring have been historically associated with the management of diabetes mellitus, a chronic metabolic disorder that affects almost 0.5 billion people worldwide^1^ and limits their capacity to maintain safe blood sugar levels.^2^ Diabetic patients require glucose monitoring devices to ensure their blood glucose remains within healthy levels (4 to 7 mM)^3^ to avoid complications, such as hypoglycemia and hyperglycemia.^4^ Recent studies have highlighted the relevance of glucose sensing beyond diabetes management. For instance, glucose levels can be a diagnostic biomarker for several diseases, including pancreatic cancer and nonalcoholic fatty liver disease (NAFLD).^5,6^ Additionally, monitoring glucose levels provides important prognostic information for patients undergoing treatment for various illnesses, such as cancers and traumatic brain injury.^7,8^ Moreover, glucose monitoring also have introduced essential benefits in other areas, such as sports medicine and personalized nutrition.^9,10^

Various sensing strategies have been developed to detect glucose, mainly following optical,^11^ colorimetric,^12^ chromatography,^13^ and electrochemical means.^14^ Among these methods, electrochemical detection integrated into disposable test strips has found extensive application in the medical field for its rapid read-out, low cost, portability, high sensitivity, and specificity.^15^ These sensors generally exploit the enzymatic properties of the protein glucose oxidase (GOx) to oxidize β-d-glucose to gluconic acid,^16^ which, when coupled with an electron transfer mediator, enables quantitative detection of glucose.^17^ GOx is usually immobilized directly onto the working electrode. However, environmental stress, such as variability in temperature, altitude (producing changes in oxygen content), air exposure, and humidity, can decrease the enzymatic activity of GOx, reducing the lifespan of test strips. To this end, the use of nanostructures for enzyme confinement has become an area of growing interest to limit drawbacks derived from environmental exposure.^18,19^

Ordered porous coatings with pores in the mesoscale (2–50 nm) made from various material matrices including carbon,^20^ gold,^21^ polymers,^22^ silica,^23^ metal-oxides,^24^ and aluminosilicates^25^ have been widely investigated for enzyme confinement due to the ability to tailor their structural parameters by molecular design.^26,27^ In particular, the use of block copolymers as structure-directing agents enables tuning the material matrix, porosity, pore size, and film thickness.^28^ Commonly used strategies for enzyme immobilization include physisorption, covalent bonding, and enzyme cross-linking.^29^ While the interaction of the enzyme with the porous structure and surface functional groups may significantly affect biosensor performance,^30^ there is, to date, no universal method for enzyme immobilization in porous materials with a view of maintaining enzyme activity, selectivity, and stability.^31,32^

GOx is a dimeric globular protein that catalyzes glucose oxidation into gluconic acid by accepting electrons. In second-generation glucose sensors, the excess electron is transferred to an electron transfer mediator (i.e., a redox probe).^17^ Consequently, the electrochemical glucose sensor performance is directly related to the enzyme loading capacity, accessibility to the active site, and the redox probe diffusion toward the working electrode.^29,33^ GOx exhibits molecular dimensions of 7.0 × 5.5 × 8.0 nm^3^ and is negatively charged at neutral pH (pI 3.9–4.3).^29^ Similarly, the aluminosilicate surface has been reported to be negatively charged at neutral pH.^34^ Therefore, repulsive effects between the pore walls and GOx can limit molecular diffusion, making it essential to consider alternatives. To this end, the selective surface modification with positively charged functions (at neutral pH) or covalent bonding may reduce electrostatic repulsion to enable GOx diffusion while preserving the enzyme in the nanostructure and minimizing leaching after immobilization.

Previous studies investigating the surface modification of porous aluminosilicates and silicates for GOx loading have primarily focused on bulk porous particles such as SBA-15 and MC-41.^35−38^ These studies generally involve coating the electrode with no preferred particle and pore orientation, making it challenging to distinguish the effect of surface modification on enzyme loading within the pores from that on the interparticle space and external surface. This lack of immobilization control within the pores presents a significant limitation for applying these materials in biosensing applications.

This work studies the effect of surface modification of ordered porous aluminosilicate-coated electrodes in enzymatic electrochemical glucose sensors. Such porous thin films were fabricated by deploying self-assembled block copolymer micelles as porogenic templates to coordinate inorganic nanoparticles into an ordered nanoarchitecture. Different strategies for GOx immobilization in the ordered porous thin films were compared in real-time using a quartz crystal microbalance with dissipation monitoring (QCM-D), namely by (a) the native oxide surface as well as after (b) amino surface modification, (c) aldehyde surface modification and (d) oxygen plasma surface activation of the native surface. Subsequently, the electrochemical activity of the porous thin films loaded with GOx was studied by cyclic voltammetry. Finally, the glucose detection performance was evaluated by chronoamperometry in concentrations relevant to medical applications. Our results highlight surface chemistry’s role in enabling biosensing applications using ordered porous thin films and provide guidelines for designing effective and durable glucose sensors.

## Results and Discussion

### Fabrication of a Porous Aluminosilicate-Coated Working Electrode

**Figure 1:**
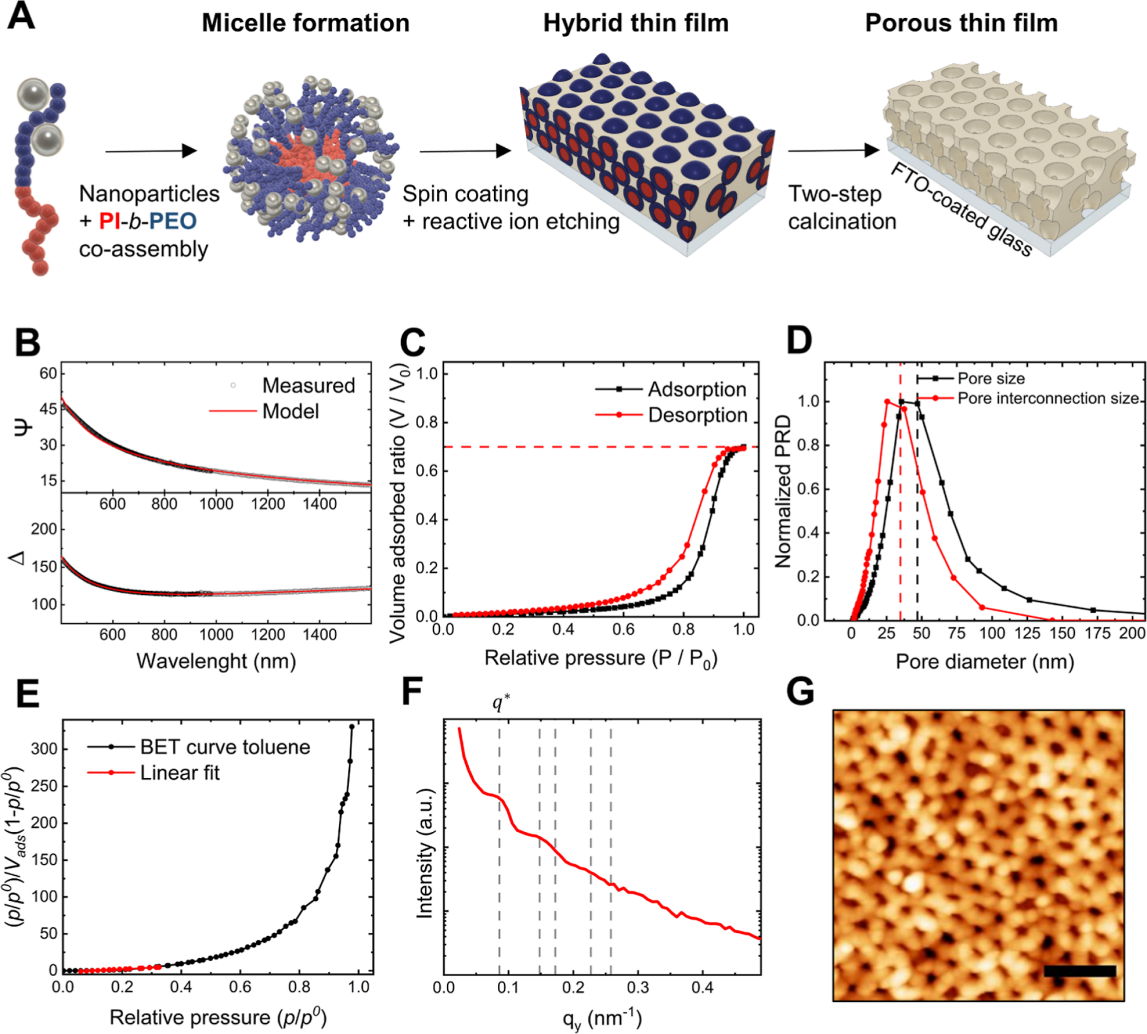
Structural characterization of porous aluminosilicate coatings. (A) Schematic of the fabrication process of porous aluminosilicate thin films. (B) Spectroscopic ellipsometry angles and corresponding fitting used to determine film thickness and refractive index. (C) Adsorption–desorption isotherms obtained by ellipsometric porosimetry. (D) Pore size and pore interconnection size distributions derived from the EP isotherms. (E) BET plot and linear fit applied to obtain the surface area. (F) In-plane line-cuts integration of the GISAXS scattering pattern with the nominal peak position ratios (*q*/q*) of 1, 1.73, 2, 2.64, and 3 for reference. (G) AFM image of a porous film surface (AFM scale bar: 250 nm).

We used sacrificial materials templating to produce inorganic porous coatings directly onto the working electrode of a three-electrode setup, as illustrated in Figure 1A. In short, we co-assembled poly(isoprene)-*block*-poly(ethylene oxide) (PI-*b*-PEO) micelles with aluminosilicate nanoparticles in solution and spin-coated them onto the working electrode, generating a thin film. The hybrid coatings were then calcined to condense the inorganic nanoparticles into a continuous matrix and to remove the block copolymer (BCP) micelles, generating pores. We used a high organic–inorganic ratio (50%), i.e., the ratio between the BCP and the nanoparticles, to produce a material with large accessible porosity and pore sizes near the upper limit of the mesoscale (i.e., 50 nm) in order to maximize enzyme loading. We chose a fluorine-doped tin oxide (FTO) coated glass as the working electrode, whose distinct thermal stability was crucial for enabling our high-temperature pore fabrication protocol while ensuring good film adhesion to the substrate. Please note that the two-step calcination process was key to generating an optimal nanoarchitecture and preserving pore dimensions and structure, as we have previously reported for this material system.^39^ Alternative methods for achieving various pore dimensions in aluminosilicates are discussed elsewhere.^40,41^

We employed spectroscopic ellipsometry to determine the film thickness and refractive index and ellipsometric porosimetry (EP) to measure the accessible porosity, pore size distribution, and surface area of the porous thin films. The film thickness and refractive index were obtained by measuring the ellipsometric angles ψ and Δ in both visible and infrared ranges, resulting in a thickness of 170 nm and a refractive index of 1.10, as represented by the red line in Figure 1B. The maximum toluene adsorption in the nanostructure revealed an accessible porosity of 70%, as indicated by the red dashed line in Figure 1C. The shape of the EP isotherm was characteristic of type IVa isotherms with an H2 (b) hysteresis loop, typical of mesoporous materials featuring wide pores connected through large pore necks, according to the IUPAC classification.^42^ The modified Kelvin equation was applied to the EP isotherm to derive a pore size distribution, which was normalized by fitting a Gaussian function (mean size ± standard deviation), yielding 46.9 ± 14.4 nm, while the pore interconnection size distribution was 34.8 ± 11.9 nm, as illustrated in Figure 1D. The BET method was applied to the porosimetric data to determine the surface area, which was found to be 103 m^2^ cm^–3^, as shown in Figure 1E.

Next, we performed grazing incidence small-angle X-ray scattering (GISAXS) on the porous thin films to elucidate the porous order (Supporting Information Figure S1). The analysis of the 2D scattering patterns (Figure 1F) revealed Bragg peaks in the in-plane integration (*q*_*y*_), indicating the presence of in-plane porous order consistent with the early formation of various symmetry groups (e.g., HCP, FCC, BCC). In addition, a pore center-to-center distance (*D*_c–c_) of 84 nm was derived from the first Bragg peak (q*). This implies that the film thickness consists of approximately six layers of pores, taking into account the usual 66% contraction of the film during the calcination process.

We captured atomic force microscopy (AFM) images on the film surface to confirm the porous features, as shown in Figure 1G. A *D*_c–c_ of 77 nm was obtained from the nearest neighbor analysis of the AFM image (see Supporting Information Figure S2). Please note that we performed reactive ion etching on the hybrid films (see etching rates in Supporting Information Figure S3A). This step was taken to prevent the formation of a superficial inorganic deposit that might impede pore accessibility (Supporting Information Figure S3B). Table 1 summarizes all the structural parameters obtained for the fabricated porous aluminosilicate thin films.

**Table 1:** Porous Parameters Obtained from Structural Characterization

calcination process	film thickness [nm]	porosity [vol %]	mean pore size *D*_ads_ [nm]	mean pore interconnections size *D*_des_ [nm]	surface area [m^2^ cm^–3^]	centre-to-centre pore distance *D*_c–c_ [nm]
two-step	170	70	46.9 ± 14.4	34.8 ± 11.9	103	84

The pore dimensions and nanoarchitecture obtained are promising for facilitating the diffusion of both small (electrolyte) and large (GOx) molecules involved in glucose sensing while providing capacity for a high enzyme loading with minimized leaching. Recent studies have demonstrated that pore shape, structure, and interconnection size are key structural factors influencing enzyme loading into porous materials.^44,45^ Accordingly, the porous network dimensions should be greater than the enzyme size to enable enzyme diffusion throughout the entire pore structure and, thus, achieve high enzyme loading.^44^ However, pore restrictions smaller than 20 nm, typically presented in the form of pore interconnections, have been found to decrease protein loading due to protein–protein repulsion that may prevent further enzyme diffusion and immobilization into the pores (for covalent bonding).^33^ Pore sizes between 50 and 70 nm have been reported as optimal for protein immobilization when comparing materials with pores ranging from 1 to 100 nm.^33^ Smaller pores exhibited a decreasing trend in enzyme loading capacity, while pore sizes above 70 nm resulted in higher rates of protein leaching.^33^ Hence, the dimensions of the pores fabricated in this study fall within the favorable range to maximize enzyme loading while minimizing its leaching.

### Glucose Oxidase Loading in Surface-Modified Porous Aluminosilicates Thin Films

We compared the porous aluminosilicate coatings with various surface chemistries (SC) aiming to store GOx in the pores, namely the native oxide surface (SC1), chemical modification with amine groups (SC2), their subsequent chemical modification resulting in aldehyde groups (SC3), and oxygen plasma activation of the native surface (SC4).

**Figure 2:**
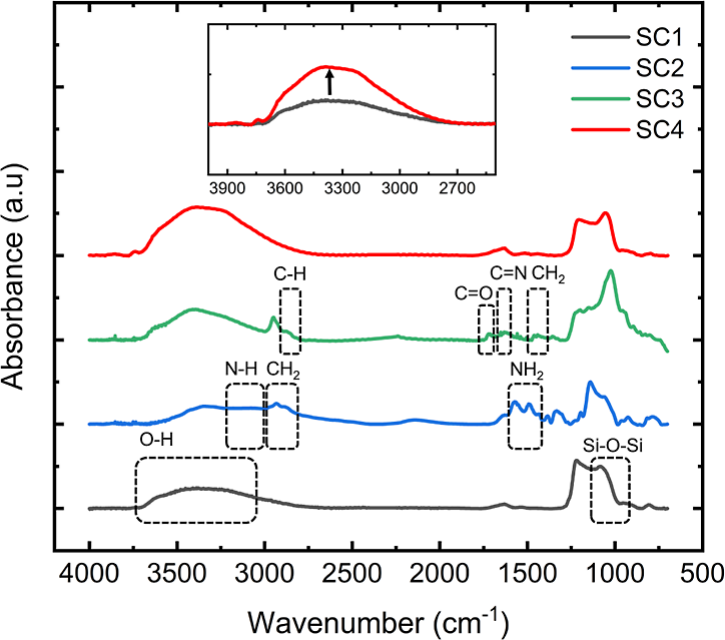
FTIR spectroscopy of surface-modified aluminosilicate porous thin films.

We measured the FTIR spectra of the surface-modified porous films to confirm the chemical modifications, as shown in Figure 2. The SC1 surface was characterized by the Si–O–Si (∼1040 cm^–1^) and O–H peaks (∼3000–3500 cm^–1^). Amino-modification in SC2 was consistent with the two characteristic peaks of NH_2_ groups (1550 and 1485 cm^–1^). The aldehyde functionalization of SC3 was evident by the presence of C=O peaks (1720 cm^–1^), the C=N bonds formed (1652 cm^–1^), and the loss of the NH_2_ peaks. FTIR spectra of the SC4 surface resembled the surface of the SC1 sensor. However, the direct comparison of the O–H band (Figure 2 inset) confirmed that the plasma treatment incorporated hydroxyl groups on the surface.

**Figure 3:**
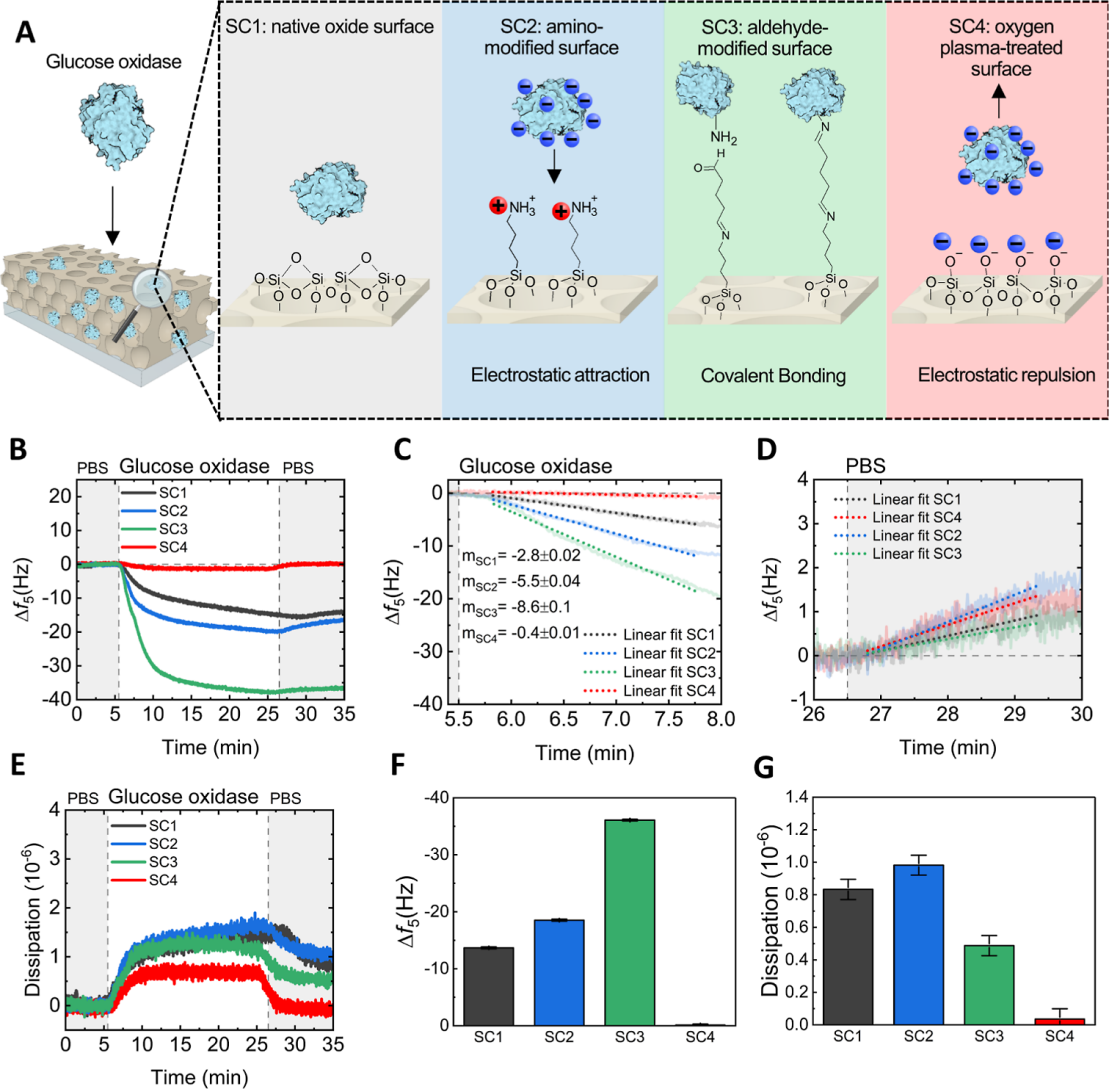
Real-time glucose oxidase immobilization into porous aluminosilicates thin films. (A) Schematic of the surface modification and enzyme interaction with the pore walls. (B) Frequency response (5th harmonic) upon glucose oxidase exposure of sensors coated with surface-modified mesoporous thin films. (C,D) First minute of adsorption and desorption of the surface-modified sensors, respectively. (E) Dissipation response of the sensors. (F,G) Summary of the surface-modified mesoporous sensors’ net frequency and dissipation changes, respectively.

Figure 3A schematizes a simplified model of the pore surface modifications and their resulting interaction with GOx. The native oxide surface of the material presents weak polarisation after high-temperature calcination, as it results in irreversible condensation of silanol (Si–O–H), leading to a relatively high density of siloxane (Si–O–Si) on the surface (and low O–H density).^44,46^ However, it should be noted that the surface is likely to remains (weakly) negatively charged at this pH due to the deprotonation of any remnant surficial silanol groups not condensed during calcination.^47^ The amino-modified aluminosilicate surface is expected to be positively charged in aqueous solutions (at neutral pH) due to protonation of the aminopropyl moieties (NH_3_^+^),^48,49^ which attractively interact with the negatively charged enzyme via electrostatic interactions. Pore walls modified with aldehyde groups may covalently bond GOx. This is achieved by reacting the amine groups of lysine residues in the enzyme with one or more aldehyde groups on the modified surface.^50,51^ Finally, oxygen plasma treatment can further increase the negative charge on the aluminosilicate surface by forming more silanol groups (high O–H density), resulting in a long-range repulsive effect on GOx due to their similar surface charge.^52^ It is relevant to note that the previous description may not apply directly to inorganic mesoporous materials fabricated with the amino modification via a one-pot method because they present a different surface charge.^53^

We then employed a quartz crystal microbalance with dissipation monitoring (QCM-D) to investigate the influence of surface chemistry on GOx loading into the porous coatings. QCM sensors coated with porous aluminosilicates were used to monitor the changes in frequency and dissipation upon GOx exposure in real time, enabling the measurement of maximum enzyme loading into the nanomaterial and the corresponding diffusion rates. The changes in frequency (5th harmonic) of QCM sensors exposed to 2 mg mL^–1^ of GOx in PBS (pH 7.4) were recorded, as shown in Figure 3B. Net negative changes in frequency were observed in sensors SC1, SC2, and SC3 after rinsing with PBS, indicating that GOx effectively diffused through the porous coating. Conversely, minor changes in frequency were found in the SC4 sensor, indicating that the repulsive electrostatic interactions restricted GOx diffusion through the pores. This suggests that surface chemistry (and charge) is crucial in immobilizing macromolecules within an ordered porous structure. Finally, we analyzed the first minute of adsorption (Figure 3C) to compare the effect of the surface chemistry on enzyme diffusion within the pores (diffusion rates). The GOx diffusion rate into the SC1 sensor (*m*_SC1_ = −2.8 ± 0.02 Hz cm^–2^ min^–1^) was found to be slower, nearly half the diffusion rate into the SC2 sensor (*m*_SC2_ = −5.5 ± 0.04 Hz cm^–2^ min^–1^), and one-third the rate in SC3 sensor (*m*_SC3_ = −8.6 ± 0.1 Hz cm^–2^ min^–1^). No significant differences in diffusion rates were observed during PBS rinsing (Figure 3D), suggesting that the ordered porous nanoarchitecture effectively minimized enzyme leaching.

The dissipation changes measured in the sensors are shown in Figure 3E (ppm, 10^–6^). Upon exposure to GOx, all sensors displayed small net dissipation changes (<1 ppm), suggesting that the enzyme immobilized within the pores exhibited a rigid behavior, which may be attributed to confinement effects produced by the pores, as reported in previous studies.^54^

Figure 3F,G summarizes the net frequency and dissipation changes observed in all porous sensors. Notably, the total frequency changes measured in the SC3 sensor are more significant than those in SC1 and SC2. This difference could be attributed to the distinct equilibrium dynamics between physisorption (SC1 and SC2) and covalent bonding (SC3; chemisorption) enzymes into pores. Enzyme diffusion is a nonequilibrium process that is induced by concentration gradients and moves toward equilibrium. Consequently, the enzyme concentration within the film increases until a balance between adsorption and desorption is achieved, i.e., when the concentration in the pores equals the solution concentration. This may explain the similar net frequency changes found between SC1 and SC2 sensors despite differences in adsorption rates. In contrast, the permanent bonding of enzymes to the pore walls in the SC3 sensor may reduce enzyme desorption, thereby reaching equilibrium at a higher enzyme loading. Finally, the minor frequency changes measured in the SC4 sensor led to discarding further testing of this approach for glucose sensing.

In summary, the use of QCM-D was effective in studying the effect of surface chemistry on enzyme loading into porous aluminosilicates thin films, as it confirmed that GOx was immobilized in a highly efficient manner within the porous nanoarchitecture via three distinct chemical routes, namely the native oxide layer, amino-modified, and aldehyde-modified surface. Additionally, the application of oxygen plasma activation provided notable results concerning the inhibitory effects of similar surface charges in limiting the diffusion of macromolecules within a porous network with nanometric dimensions. Finally, it is worth noting that QCM-D enables to gain new insights regarding enzyme adsorption dynamics (e.g., adsorption/desorption rates, timescale) in mesoporous materials.

### Electrochemical Glucose Detection Using Surface-Modified Porous Aluminosilicates

**Figure 4:**
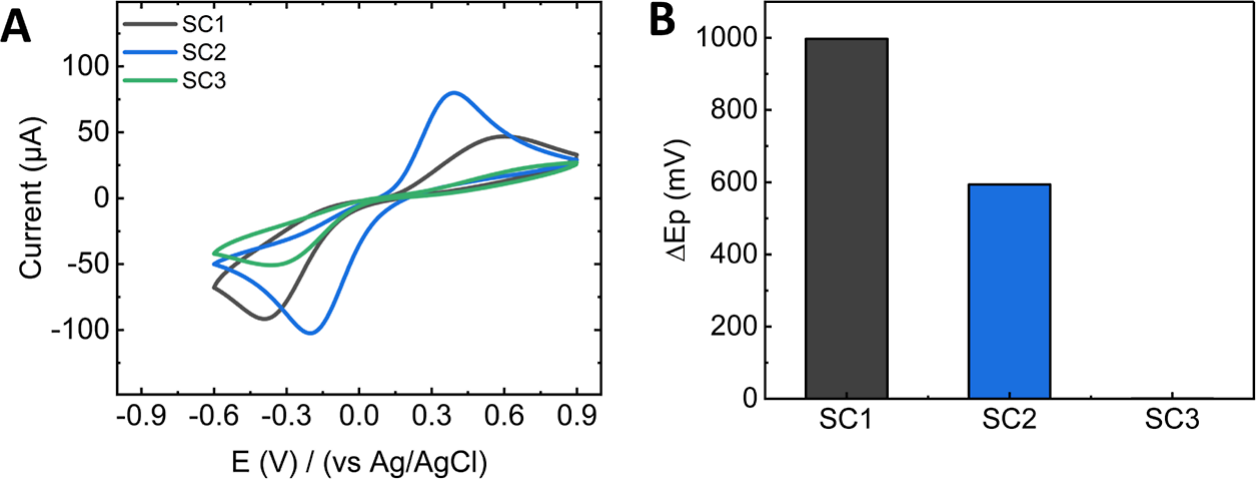
Electrochemical activity of electrodes coated with surface-modified porous thin films. (A) Cyclic voltammogram of the porous electrodes in ferricyanide (scan rate: 100 mV s^–1^). (B) Summary of the peak-to-peak separation measured in the CVs.

Initially, to characterize the electrochemical activity of the SC1, SC2, and SC3 sensors, a cyclic voltammetry (CV) analysis was conducted on the GOx-loaded porous films using the negatively charged redox probe potassium ferricyanide (2 mM), which is commonly employed for glucose sensing,^55^ as depicted in Figure 4A.

We found that the SC1 sensor exhibited two barely defined redox peaks at 603 mV for oxidation and −394 mV for reduction with a peak-to-peak separation (Δ*E*_p_) of 997 mV (Figure 4B SC1) and an anodic/cathodic ratio (*i*_an_/*i*_cat_) of 0.44. The observed irreversibility of the ferricyanide redox reaction in the unmodified porous coating may be attributed to aluminosilicates’ net negative surface charge due to residual silanol groups not being incorporated into the siloxane network during calcination. This negative surface charge, coupled with the high ionic strength, produces a compact Debye layer that partially opposes the diffusion of negatively charged molecules such as ferri/ferro ions.^56^ Moreover, the repulsive effect of the packed GOx in the porous film may further reduce the effective percolation paths for ferricyanide, leading to the observed irreversibility. We identified an improved electrochemical response in the SC2 sensor, which exhibited two better-defined redox peaks (Figure 4A blue line). The oxidation peak was found at 392 mV and the reduction peak at −202 mV, resulting in an Δ*E*_p_ of 594 mV and an anodic/cathodic ratio *i*_an_/*i*_cat_ of 0.7. These results suggest that amino modification facilitates the diffusion of small molecules within the pore structure, leading to a more reversible redox reaction than the native oxide layer. In contrast, the CV of the SC3 sensor did not exhibit two characteristic redox peaks in the potential range studied (Figure 4A green line). This negligible electrochemical activity may be related to enzymes blocking the percolation paths toward the working electrode, thus, impeding the exchange of charge carriers. We ensured that the lack of electrochemical response was not a consequence of the chemical modification procedure by measuring the CV of a bare FTO glass coated with GOx following the same protocol for SC3 (see Supporting Information Figure S4). The oxidation and reduction peaks observed suggest that GOx bound to the bare electrode did not block access to the conductive working electrode. Furthermore, previous studies suggest that this functionalization protocol retains the catalytic activity of GOx on mesostructured surfaces while also preserving the electrochemical response of porous nanoparticles.^36,57^ These findings indicate that a large amount of GOx covalently bound to an ordered porous structure may hinder diffusion-related applications that require access to the underlying substrate.

The absence of electrochemical response observed in the case of covalently immobilized GOx on the pore walls precluded further testing for glucose detection with the SC3 sensor. Thus, glucose detection was optimized for the SC2 sensor using an operating potential that surpasses the oxidation potential measured for ferrocyanide oxidation in this sensor (+0.6 V vs Ag/AgCl). The unmodified-porous thin film SC1 served as a reference.

**Figure 5:**
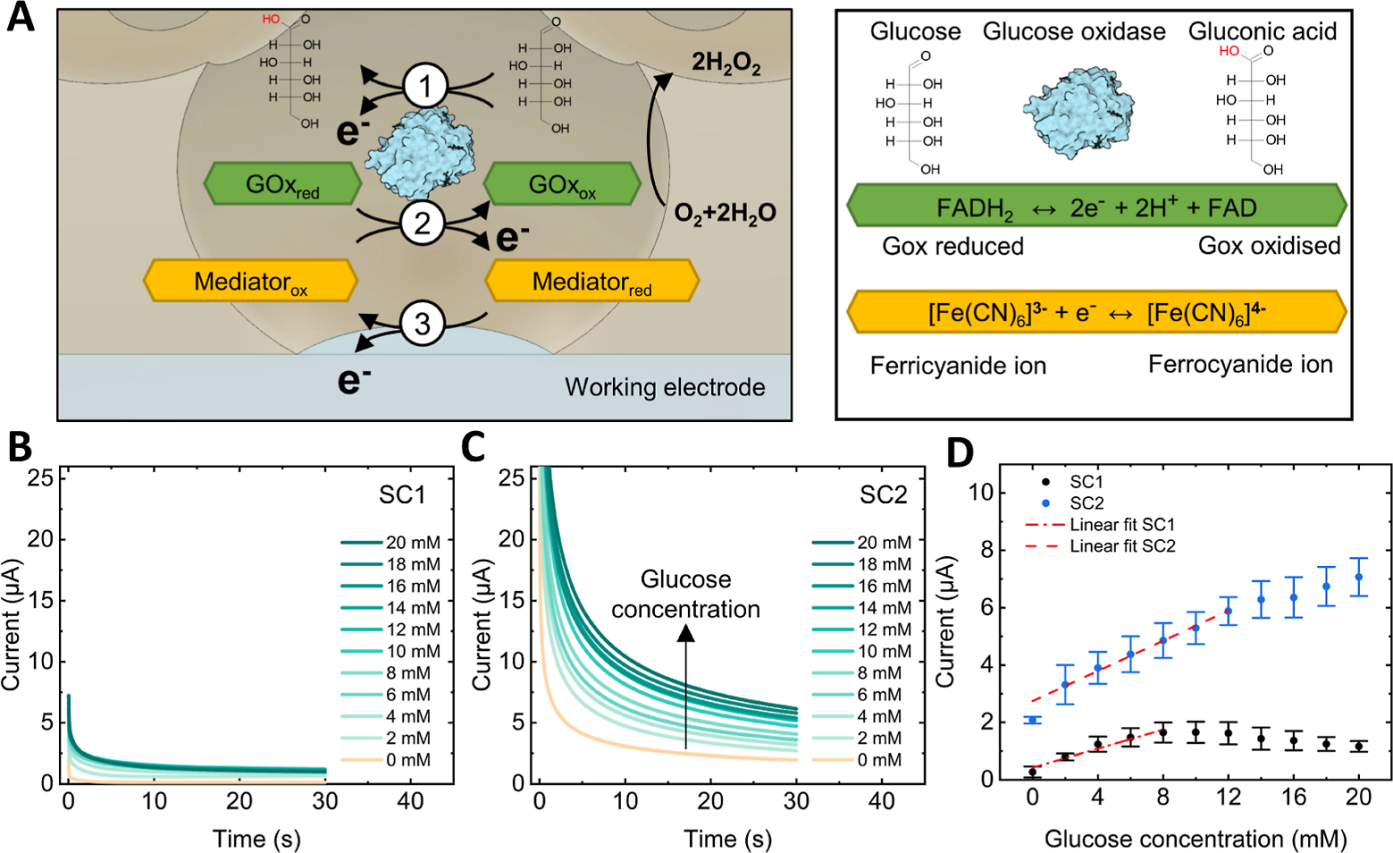
Electrochemical detection of glucose. (A) Schematic of the electrochemical detection of glucose in the pores using glucose oxidase and an electron transfer mediator. Chronoamperometric measurements with increasing glucose concentrations (2–20 mM) using the (B) SC1 porous sensor and (C) SC2 porous sensor (potential: +0.6 V vs Ag/AgCl). (D) Glucose concentration–response curves obtained with the SC1 and SC2 porous sensors. The error bar corresponds to the standard deviation of three measurements.

We studied the electrochemical detection of glucose in concentrations relevant to clinical applications by chronoamperometry using the modified porous transducers loaded with GOx (working electrode). Figure 5A schematizes the electrochemical reactions involved in the enzymatic detection of glucose inside the pores. Briefly, once glucose is added to the sensor, the active site of GOx, flavin adenine dinucleotide (FAD), acts as a catalyst to oxidize glucose (β-d-glucose) into glucono δ-lactone (d-glucono-1,5-lactone),^58^ which then hydrolyzes into gluconic acid in aqueous solution with hydrogen peroxide being produced as a by-product.^59^ In the catalytic reaction, FAD accepts two electrons and is reduced to FADH_2_ (1 in Figure 5A),^60,61^ which is then oxidized to FAD by transferring electrons to the electron transfer mediator, ferricyanide. Consequently, ferricyanide is reduced to ferrocyanide, and FAD is available to oxidize another glucose molecule (2 in Figure 5A).^62^ Finally, a fixed potential triggers the oxidation of ferrocyanide into ferricyanide at the electrode interface, releasing an electron in the process, which is collected by the working electrode (3 in Figure 5A). Accordingly, the quantity of electrons measured by the working electrode is proportional to the amount of glucose in the solution, enabling glucose quantification.

In diabetic individuals, hypoglycemia and hyperglycemia arise when glucose levels decrease below 3.2 mM (70 mm/dL) or rise beyond 10 mM (180 mm/dL), respectively.^3,4^ Accordingly, chronoamperometric data from a 2 mM stepwise increase of glucose concentrations between 2 and 20 mM for the SC1 and SC2 porous sensors are shown in Figure 5B,C, respectively. Minor changes in the oxidation current were observed in the SC1 sensors (Figure 5B). In contrast, the current measured in the SC2 sensors increased proportionally for similar glucose concentrations (Figure 5C).

Figure 5D shows the current response obtained after 30 s for glucose concentration from 2 to 20 mM deploying sensors SC1 and SC2, respectively. The oxidation current measured using the SC1 sensor increased linearly with glucose concentration between 2 and 8 mM (*n* = 3). Additional increments in glucose did not further increase the oxidation current. Equation 1 shows the linear fit (*R*^2^ = 0.925) in the 0 to 8 mM range. In the blank, i.e., at 0 mM glucose concentration, these sensors exhibited an average response of 0.27 μA, with a standard deviation of 0.19 μA.
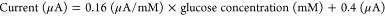
1

The SC2 sensors (*n* = 3) displayed a linear increase in oxidation currents correlating with the glucose concentration from 2 to 14 mM. Equation 2 corresponds to the linear fit (*R*^2^ = 0.975) of this relationship that allows glucose quantification within this range. For these sensors, the mean response observed in the blank was 2.07 μA, with a standard deviation of 0.12 μA.
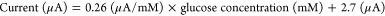
2

In the linear regression analysis, the amino-modified SC2 porous sensors demonstrated an enhanced glucose sensitivity (0.26) compared to the unmodified SC1 sensor (0.16). Furthermore, the limit of detection, determined as three times the standard deviation of the blank divided by sensitivity, was calculated to be 3.6 mM for SC1 and 1.4 mM for SC2. Most importantly, the concentration range that the amino-modified sensors can detect glucose is relevant for clinical applications.^3^ These findings for ordered porous thin films differ from earlier studies focused on unmodified bulk porous particles, where GOx immobilization and electrochemical glucose detection performance were directly correlated.^63,64^

In summary, covalently binding GOx to the pore walls resulted in negligible electrochemical activity, which we attributed to the enzyme permanently blocking the pore interconnections and, thereby, the access to the substrate. Loading the pores with GOx using the native oxide material was possible. However, the electrostatic repulsion between aluminosilicate pore walls, GOx, and ferricyanide was detrimental to sensing. On the contrary, the amino-modified porous sensor showed greatly improved electrochemical activity, indicating that selective surface modification could enhance the diffusion of both large (enzyme) and small (electrolyte ions) molecules through inorganic porous films. Therefore, designing an inorganic porous material platform for biosensing applications using enzymes should consider the effects of electrostatic interactions between large and small molecules and the pore walls and the blocking effect that covalent binding presents. These findings are relevant not only to glucose sensing but also to similar systems that may use charged macromolecules in inorganic porous materials, such as nucleic acids (e.g., DNA and RNA) and other enzymes, such as CRISPR Cas9 used for gene editing.^65,66^

## Conclusions

In conclusion, our study highlights the critical role of surface modification of ordered porous materials for electrochemical biosensing. We coated a conductive oxide with an ordered porous aluminosilicate film of ∼170 nm thickness, a pore diameter of ∼50 nm, and a pore interconnection size of ∼35 nm and applied various modification protocols to study the effect of electrostatic attraction (amino functionalization), covalent bonding (aldehyde functionalization) and electrostatic repulsion (oxygen plasma treatment) on the immobilization of glucose oxidase and its functioning in electrochemical glucose sensing. We demonstrate that surface modification with positively charged molecules facilitates the integration of negatively charged enzymes and electrolytes in otherwise unfavorable conditions. Specifically, amino-modified porous aluminosilicates displayed better sensitivity and a wider linear range for electrochemical detection of glucose compared to unmodified porous architectures. On the other hand, aldehyde functionalization showed pronounced enzyme uptake but only limited electrochemical activity for glucose-sensing applications. In consequence, translating the enzymatic properties of glucose oxidase toward quantitative glucose detection in conditions relevant to clinical applications was only possible with the amino modification.

Our findings emphasize the importance of supramolecular chemistry when designing electrochemical biosensors with nanometric features. Moreover, this work offers guidelines for other applications of porous networks, where surface modifications for electrostatic balancing may be equally important. As an example, the introduction of negative charges in order to favor the storage of positively charged enzymes may help to store CRISPR-Cas9 enzymes used for gene editing, which have a hydrodynamic radius of ∼7 nm and are positively charged at neutral pH.^67^

## Experimental Section

### Reagents

All reagents were used as received without further purification. Block copolymer poly(1,4-isoprene)-*block*-poly(ethylene oxide) PI-*b*-PEO (polydispersity: 1.01, Mn PI: 48, PEO: 12 kg mol^–1^) was obtained from Polymer Source. The following reagents were purchased from Merck: glucose oxidase from *Aspergillus niger* (type X–S, lyophilized powder, 100.000–250.000 units g^–1^ solid without added oxygen), d-(+)-glucose (≥99.5% (GC)), 1-butanol (99.4%), toluene (99.9%), toluene (anhydrous, 99.8%), (3-glycidyloxypropyl)-trimethoxysilane (GLYMO) (≥98%), aluminium tri-*sec*-butoxide (97%), potassium chloride (KCl) (≥99.9%), (3-aminopropyl)triethoxysilane (APTES) (99%), glutaraldehyde solution (Grade I, 25% in H_2_O, specially purified for use as an electron microscopy fixative) and ethanolamine hydrochloride (≥99.0%). Electrolytes potassium ferricyanide (99+%, K_3_[Fe(CN)_6_]) and potassium ferrocyanide (>98.5%, K_4_[Fe(CN)_6_]) were purchased from ACROS Organics and Honeywell, respectively. Phosphate-buffered saline (PBS) tablets were obtained from OXOID.

### Fabrication of the Mesoporous Transducer

#### Aluminosilicate Stock Solution Preparation

An aluminosilicate stock solution was prepared as reported elsewhere.^41^ In brief, mixing and stirring in an ice bath 0.32 g of aluminum tri-*sec*-butoxide, 2.8 g of GLYMO, and 20 mg of KCl. After 15 min of stirring, 0.135 mL of a 10 mM HCl solution was added dropwise to start the hydrolysis of the precursors and left for another 15 min in the ice bath. The mixture was then removed from the ice bath and stirred at room temperature for 15 min. 0.85 mL of 10 mM HCl was added to the solution and stirred for 20 min to complete the hydrolysis. The final solution was filtered with a 0.2 μm cellulose syringe filter and dissolved with 2.135 mL of toluene/1-butanol (72.84/27.16 wt %) to obtain 1 g mL^–1^ of aluminosilicate. The mixture was then kept refrigerated at 5 °C prior to use.

#### Fabrication of Porous Aluminosilicate Thin Films by Block Copolymer Co-assembly

40 mg mL^–1^ of the BCP PI-*b*-PEO was dissolved in an azeotropic mixture of toluene/1-butanol (BCP stock solution). Next, 60 μL of the aluminosilicate stock solution was mixed with 0.5 mL of the BCP stock solution in a glass vial and mixed in a shaker for 30 min before use. 40 μL of the prepared solution was subsequently dispensed on flat substrates and spin-coated (2000 rpm, 20 s, Laurell WS 650 MZ) to form thin films. Thereafter, samples were reactive ion etched in CHF_3_ (CHF_3_/Ar 15/50 sccm, 2 min, 215 W, 40 mbar, PlasmaPro 80 RIE, OXFORD instruments). The thin films were calcined under inert conditions in a tubular furnace (450 °C, Ar, 30 min, 5 °C min^–1^) and left to cool inside the furnace. Finally, thin films were calcined in air (450 °C, 30 min, 5 °C min^–1^). Please note that calcination in inert conditions prior to air calcination was necessary to obtain an adequate porous structure with good mechanical stability.^68^

#### Surface Modification of the Porous Sensors

Porous aluminosilicates with different surface modifications were prepared before GOx immobilization, labeled as SC1, SC2, SC3, and SC4. No surface modification was used for SC1 sensors. SC2 sensors were modified with APTES. SC3 sensors were first modified with APTES and subsequently with glutaraldehyde. SC4 sensors were oxygen plasma modified (300 s, 100 W, 0.33 mbar, Diener Electronic “Pico”).

APTES modification was performed under an argon atmosphere by immersing the sensors for 20 min in 5% v/v of APTES in anhydrous toluene. The sensors were consecutively sonicated in toluene (2 × 5 min) and ethanol (1 × 5 min) to remove unreacted APTES.

After APTES functionalization, the sensors used for covalent bonding (SC3) were immersed for 30 min in a 10% v/v glutaraldehyde in 0.1 M PBS buffer. Next, the sensors were sonicated in PBS (2 × 5 min) to remove unreacted glutaraldehyde molecules.

#### Glucose Oxidase Immobilization on the Porous Sensors

Surface-modified sensors used for electrochemical sensing were immersed overnight in a 2 mg mL^–1^ GOx in 0.1 M PBS solution (pH 7.3). The sensors were subsequently washed with 0.1 M PBS to remove GOx that was not immobilized in the nanostructure.

### Material Characterization

#### Substrates

Material characterization and electrochemical detection of glucose were performed using the following substrates: fluorine tin oxide coated glass (20 × 15 mm^2^, TEC 6, Pilkington) served as the working electrode for glucose detection. Si-coated QCM sensors (5 MHz, 14 mm Cr/Au/SiO_2_, Quartz PRO) were used in QCM measurements. The polished side of single-side polished Si substrates (10 × 10 mm, p-type boron, MicroChemicals) was used for AFM, SEM, SE, EP, and GISAXS characterization. Au-coated (100 nm, E306A Bell Jar Thermal Evaporator, Edwards) Si substrates (10 × 10 mm^2^) served for FTIR measurements.

#### Spectroscopic Ellipsometry and Ellipsometric Porosimetry

SE and EP were measured on thin films fabricated onto silicon substrates with an ellipsometer (angle: 73°, wavelength: 400–1600 nm, SE2000, Semilabs). Experimental data (Ψ and Δ) was analyzed with the integrated SEA software (Semilabs). A Cauchy dispersion law and Levenberg–Marquardt algorithm (LMA, *R*^2^ > 0.95) were fitted to the experimental data to obtain the refractive index and film thickness. Adsorption and desorption isotherms were obtained by fitting a Lorentz–Lorentz effective medium approximation (simplex fitting, tol 1 × 10^–6^, 1000 iterations) to changes in refractive index due to toluene adsorption. Pore size and pore interconnection size distribution were derived from the adsorption and desorption isotherms using a modified Kelvin equation,^69^ respectively. The contact angle between aluminosilicate and toluene was assumed to be zero (perfect wetting).^70^ A toluene cross-sectional area of 0.343 nm^2^ was employed to determine the surface area.^71^

#### Grazing-Incidence Small-Angle Scattering

2D GISAXS scattering pattern of porous films coated on a Si substrate was recorded in a Ganesha 300XL (incident angle: 0.2°, Xenocs SAXSLAB) using a high brilliance microfocus Cu-source (λ = 1.5418 Å). A PILATUS 300 K solid-state photon-counting detector (sample-to-detector distance of 950 mm) was used. FitGISAXS^72^ software was used for integration and analysis.

#### Atomic Force Microscopy

AFM images were captured in tapping mode with an AFM instrument (Dimension Icon, Bruker) using an AFM probe (nominal tip radius: 2 nm, Bruker ScanAsyst Air).

#### Scanning Electron Microscopy

SEM image in the Supporting Information was captured with an Xbeam 540 FIB/SEM (ZEISS) on a porous film without applying a metallic coating, using low acceleration voltage (0.8 kV) and 1.7 mm working distance.

#### Fourier Transform Infrared Spectroscopy

FTIR spectra were measured on surface functionalized porous thin films fabricated onto Au-coated silicon substrates using an infrared microscope (reflection mode, AIM-900, Shimadzu) with an FTIR spectrophotometer (IRTracer-1000, Shimadzu). The software Lab Solution IR (Shimadzu) was employed for CO_2_ correction and baseline adjustment.

#### Quartz Crystal Microbalance

Enzyme immobilization into the porous aluminosilicate coating was studied with a quartz crystal microbalance with dissipation monitoring (Q-Sense E4 instrument, Biolin Scientific) using a previously coated QCM sensor (5 MHz, 14 mm Cr/Au/SiO_2_, 0.79 cm^2^ active area, Quartz PRO). Solutions were pumped at a flow rate of 30 μL min^–1^ into the QCM chamber. QCM analysis (frequency and dissipation) of the harmonics *f*3, *f*5, *f*7, *f*9, *f*11, and *f*13 was performed with the software QSense Dfind (Biolin Scientific).

### Glucose Detection

#### Electrochemical Measurements

All electrochemical measurements were performed in a three-electrode setup using a silver/silver chloride reference electrode (4 mm diameter, Gamry), a platinum wire counter electrode (0.4 mm diameter, Gamry), and a porous-coated working electrode (0.5 cm^2^, FTO coated glass) containing GOx. All electrodes were assembled using an in-house built Teflon cell (see Figure S5 in Supporting Information for a schematic of the setup). Cyclic voltammetry was measured using 2 mM ferricyanide in 0.1 M PBS buffer (pH 7.3) between −0.6 and 0.9 V at a scan rate of 100 mV s^–1^. Three CV cycles were measured. Chronoamperometry (0.6 V vs *E*_ref_) and CV were measured using a potentiostat (Reference 600+, Gamry). The software Gamry Echem Analyst was used to analyze all measurements.

#### Electrochemical Detection of Glucose

Working electrodes coated with the surface-modified porous films loaded with glucose oxidase were mounted in the electrochemical cell. Then, 490 μL of 2 mM potassium ferricyanide (K_3_[Fe(CN)_6_]) in 0.1 M PBS buffer (pH 7.3) was added to the cell. Glucose detection from 0 to 20 mM was performed by infusing glucose stepwise (10.4, 10.8, 11.1, 11.93, 12.27, 12.8, 13.4, 14, and 14.64 μL) from a stock solution (100 mM in PBS) prepared the day before to allow α-d-glucose and β-d-glucose to equilibrate in solution. Glucose was infused using a syringe (1 mL, Hamilton) mounted on a syringe pump (rate: 2.651 mL min^–1^, Sigma 1110, Chronus) and connected to the electrochemical cell using Teflon tubing (0.8 mm internal diameter). Chronoamperometric measurements were performed immediately after adding glucose to the solution at room temperature and without stirring. All experiments were conducted in triplicate to account for potential variations during glucose dispensing and the subsequent measurement.

## Supporting Information Available

GISAXS 2D scattering pattern of the mesoporous film; nearest neighbor analysis; parameter optimization of the reactive ion etching process; cyclic voltammetry of bare FTO-coated film; schematic of the electrochemical cell configuration (PDF)
