# Supplementary material for: Glucose Oxidase Loading in Ordered Porous Aluminosilicates: Exploring the Potential of Surface Modification for Electrochemical Glucose Sensing
Source: Chem Mater. 2023 Sep 11;35(18):7577–87. doi: 10.1021/acs.chemmater.3c01202 (PMC10536975; doi:10.1021/acs.chemmater.3c01202)
Supplement: Supplementary file 1 — cm3c01202_si_001.pdf [file cm3c01202_si_001.pdf]

SUPPORTING INFORMATION

Glucose Oxidase Loading in Ordered Porous Aluminosilicates: Exploring the Potential of Surface Modification for Electrochemical Glucose Sensing

Maximiliano Jesus Jara Fornerod[a], *Alberto Alvarez-Fernandez*[a], *Martyna Michalska*[b], *Ioannis Papakonstantinou*[b], *Stefan Guldin*[a]*

[a]Department of Chemical Engineering, University College London, Torrington Place, London, WC1E 7JE, UK.

[b]Department of Electronic & Electrical Engineering, University College London, Torrington Place, London, WC1E 7JE UK

*corresponding author. Email address: s.guldin@ucl.ac.uk

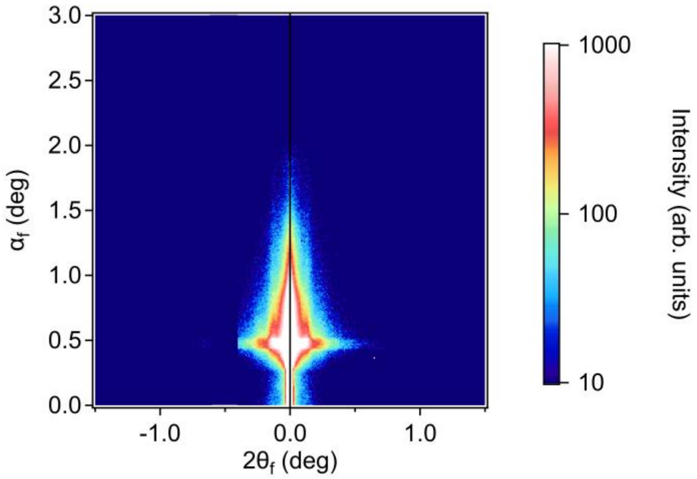

Figure S1: **GISAXS**. 2D scattering pattern of characteristic porous film.

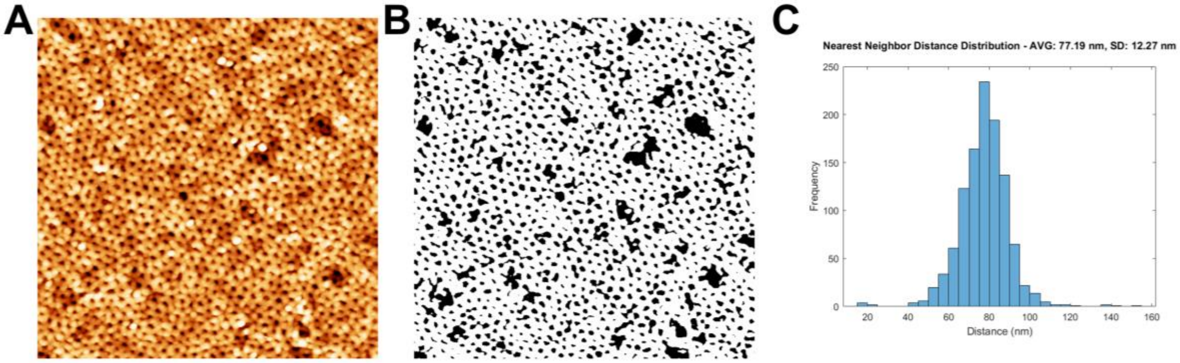

Figure S2: **Nearest neighbor analysis**. A) AFM image used for analysis (image width: 3 µm). B) Image processing to visualize pores. C) Nearest neighbor distance distribution.

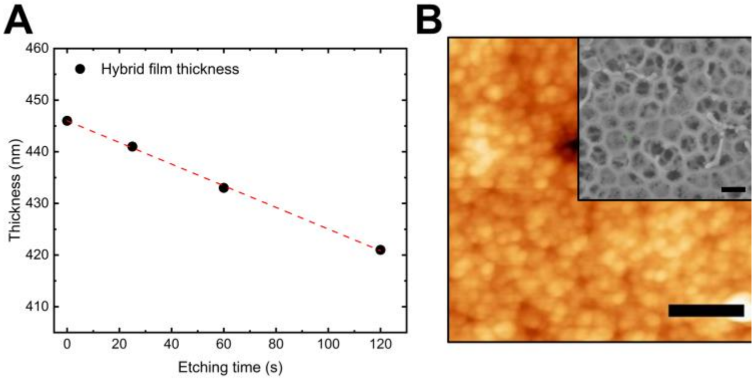

Figure S3: **Reactive ion etching of hybrid films**. A) Thickness profile measured by spectroscopic ellipsometry of hybrid films etched with CHF_3 at different times. B) AFM image of a thin film fabricated without reactive ion etching alongside a characteristic high-resolution SEM image (inset) showing the upper layer of aluminosilicate nanoparticles. (AFM scale bars: 250 nm, SEM scale bar: 50 nm).

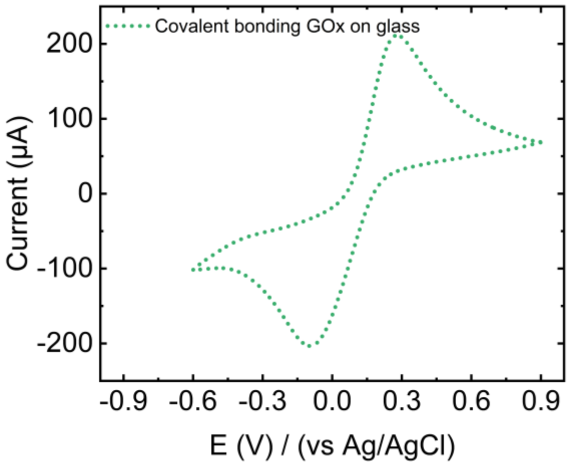

Figure S 4: **Cyclic voltammetry of bare FTO-coated glass**. CV in ferricyanide (scan rate: 100 mV s^{-1}) of an FTO-coated glass covalently modified with glucose oxidase.

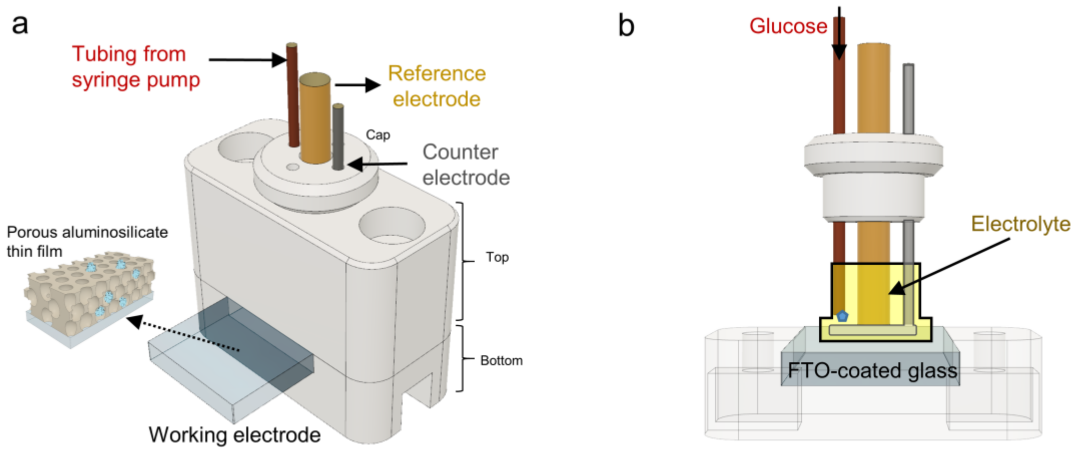

Figure S 5: **Electrochemical cell configuration**. A) Schematic of the electrochemical cell made of PTFE with a 3-electrode configuration used to perform glucose detection experiments. B) Cross-section view of the electrochemical cell.
